# Disposition of linezolid or daptomycin in Enterococcal bloodstream infections according to vancomycin resistant *Enterococcus* colonization

**DOI:** 10.1186/2047-2994-3-37

**Published:** 2014-12-01

**Authors:** Elizabeth Short, John Esterly, Michael Postelnick, Jeannie Ong, Milena McLaughlin

**Affiliations:** Department of Pharmacy, Northwestern Memorial Hospital, 251 E Huron St, Chicago, IL 60611 USA; Department of Pharmacy Practice, Chicago State University College of Pharmacy, 9501 South King Dr, Chicago, IL 60628 USA; Chicago College of Pharmacy, Midwestern University, 555 31st St, Downers Grove, IL 60515 USA; Department of Pharmacy Practice, Midwestern University Chicago College of Pharmacy, 555 31st St, Downers Grove, Downers Grove, IL 60515 USA

**Keywords:** VRE, Enterococcus, Vancomycin-resistant *Enterococcus*, Linezolid, Daptomycin, De-escalation

## Abstract

Vancomycin resistant *Enterococcus* (VRE) colonized patients are likely to receive VRE targeted Gram-positive antibiotics and may not be de-escalated appropriately once final cultures are available. A retrospective cohort study was conducted in VRE-colonized and non-VRE colonized patients with Enterococcal bloodstream infections. Of 101 patients (n = 50 VRE-colonized; n = 51 non-colonized), empiric therapy with linezolid or daptomycin was started more often in VRE-colonized than non-colonized patients (n = 8, 15.5% vs n = 27, 54%, p < 0.01). There was no difference in de-escalation once VRE infection was ruled out (non-colonized, n = 2, 66.7% vs VRE-colonized, n = 2, 50%, p = 0.09). This study encourages continued stewardship vigilance to decrease inappropriate antibiotic use.

## Background

*Enterococcus* is a common cause of nosocomial infections, and the incidence of vancomycin resistant *Enterococcus* (VRE) infection continues to rise [1, 2]. Patients with VRE infection incur an increased cost of care of $27, 190 compared to patients with vancomycin-susceptible *Enterococcus* (VSE) infection [3]. Additionally, patients with an Enterococcal bloodstream infection (BSI) who are VRE-colonized frequently receive empiric Gram-positive antibiotics targeted against VRE, such as linezolid or daptomycin. Finalized cultures growing VSE may warrant appropriate antibiotic de-escalation, which is a critical stewardship issue. Unnecessary linezolid and daptomycin use has been associated with increasing emergence of linezolid and daptomycin resistant VRE strains and represents a significant public health problem [4, 5]. We sought to determine the percent of patients with an Enterococcal BSI initially started on linezolid or daptomycin whose therapy was de-escalated once finalized culture data is available.

## Methods

A retrospective cohort study was conducted at Northwestern Memorial Hospital in Chicago, IL. Patients >18 years of age with at least one positive blood culture for *Enterococcus* spp*.*, identified between January 1, 2010 and December 31, 2011, that was treated as an active infection by the attending physician were evaluated for inclusion in the study. Patients were excluded if the attending physician elected not to treat the positive blood culture as an active infection or if the patient had a polymicrobial BSI. Only the first positive blood culture per patient during the study period was included. VRE colonization status was determined by active surveillance or previous documented VRE infection. Active surveillance is employed via rectal swab upon admission and then weekly in high-risk units including intensive care (medical and surgical), hematology and oncology, and transplant (solid organ and bone marrow) per the infection control and prevention departmental protocol. The Northwestern University and Midwestern University Institutional Review Boards approved this study.

Patients were stratified into two groups, VRE-colonized and non-VRE colonized. The primary outcome studied was the percent of patients with an Enterococcal BSI initially started on linezolid or daptomycin whose therapy was de-escalated once VRE infection was ruled out. The secondary endpoints included the percent of BSI caused by VRE, percent of patients with neutropenia, and incidence of sepsis and death. Continuous variables were evaluated with the Student’s t-test, and categorical variables were evaluated with the Chi-square or Fisher’s Exact test as appropriate. All tests were two tailed with p < 0.05 considered significant. Data analysis was performed using Intercooled Stata, version 12 (StataCorp LP, College Station, TX).

## Findings

A total of 162 patients were identified and considered for inclusion from January 1, 2010 to December 31, 2011. Of the 162 patients, 61 patients did not meet inclusion due to: polymicrobial BSI (73%), the attending physician did not treat the positive blood culture as an active infection (18%), age under 18 years (5%), and culture determined to be contamination (4%). Therefore, 101 patients were included in the analysis (n = 50 VRE-colonized; n = 51 non-colonized). VRE-colonized patients were younger than non-colonized patients (57.8 years vs. 66.1 years, p < 0.01). There were no differences in gender, race, infection source, or the place from which the patient was admitted to the hospital (p > 0.05 for all). Empiric therapy with linezolid or daptomycin was started more often in VRE-colonized than non-colonized patients (54%, n = 27 vs. 15.5%, n = 8; p < 0.01; Table 1). There were eleven non-colonized patients with VRE BSI, eight of which were initiated on VRE therapy before susceptibilities and 42 VRE-colonized patients with VRE BSI, 27 of which were initiated on VRE therapy before susceptibilities. There were eight non-colonized patients who empirically received linezolid or daptomycin, and two of the three patients that could be de-escalated were de-escalated. There were 27 colonized patients that empirically received linezolid or daptomycin and two of the four that could be de-escalated were de-escalated. For the primary outcome, there was no difference between the groups in de-escalation once VRE was ruled out (50%, n = 2 in the colonized group vs. 66.7%, n = 2 in the non-colonized group; p = 0.99). VRE-colonized patients were more likely to have VRE BSI (p < 0.01), neutropenia (p < 0.01), or a transplant (p < 0.01). There was no difference between the groups regarding sepsis (p = 0.09) and mortality (p = 0.78).

**Table 1:** **Primary and secondary outcomes of patients with enterococcal BSI**

	Non-colonized	VRE-colonized	p-value
	n = 51	n = 50	
**Primary outcomes**			
Empiric linezolid or daptomycin (n, %)	8 (15.6)	27 (54)	<0.01
Could be de-escalated (n, %)	3 (37.5)	4 (14.8)	0.31
De-escalated (n, %)	2 (66.7)	2 (50)	0.09
**Secondary outcomes**			
VRE BSI (n, %)	11 (21.5)	42 (84)	<0.01
*E. faecium* (n, %)	17 (33.3)	42 (84)	<0.01
Neutropenia (n, %)	3 (5.9)	16 (32)	<0.01
Transplant (n, %)	7 (13.7)	19 (38)	<0.01
Sepsis (n, %)	19 (37.2)	27 (54)	0.09
Mortality (n, %)	10 (19.6)	11 (22)	0.78

## Discussion

Our study found that the incidence of VRE BSI was significantly greater in the VRE-colonized group compared to the non-colonized group. While approximately half of the VRE-colonized patients were empirically started on linezolid or daptomycin, only 50% of those eligible were appropriately de-escalated. This poses a potential risk for antibiotic resistance and supports the need for continued stewardship vigilance. Inappropriate use of antibiotics with VRE activity may perpetuate resistance; therefore appropriate de-escalation is critical [6].

Our study supports reports that patients with an Enterococcal BSI with neutropenia or a transplant who are VRE-colonized are at a higher risk of VRE infection than non-colonized patients [7, 8]. Yeh et al found that patients with severe illness, prolonged hospitalization, surgical exploration, and frequent use of antibiotics are at high risk for VRE infections [9]. Empiric linezolid or daptomycin for Enterococcal BSI in these patients may be therefore warranted until VRE is ruled out; however, sepsis was not a risk factor for VRE infection.

Limitations for this study should be acknowledged. This was a retrospective cohort study and is subject to the bias associated with this study design. The study was limited to a single institution with small number of patients eligible for de-escalation; however, these stewardship issues are likely to be generalizable to other institutions.

VRE-colonized patients were more likely to be empirically stared on linezolid or daptomycin to cover VRE. This study encourages continued stewardship vigilance to decrease inappropriate antibiotic use. Further study is warranted to address this important antimicrobial stewardship issue.

## Abbreviations

BSI: Bloodstream infection
VRE: Vancomycin resistant *Enterococcus*
VSE: Vancomycin susceptible *Enterococcus*.
